# Wegener's granulomatosis and differential diagnosis of mucosal leishmaniasis

**DOI:** 10.1002/ccr3.4280

**Published:** 2021-06-23

**Authors:** Jesús Rojas‐Jaimes, Víctor Hugo Rojas‐Figueroa, Rodrigo Corcuera, José Arenas, Julio García‐Reynoso

**Affiliations:** ^1^ Faculty of Health Sciences Universidad Privada del Norte Lima Perú; ^2^ Otolaryngology Service at Hospital Guillermo Almenara Irigoyen Lima Perú; ^3^ School of Human Medicine Universidad Científica del Sur Lima Perú; ^4^ Surgical Pathology Service at Hospital Guillermo Almenara Irigoyen Lima Perú; ^5^ Rheumatology Service Hospital Nacional Arzobispo Loayza Lima Perú

**Keywords:** autoimmune disease, differential diagnosis, granulomatosis with polyangiitis, mucosal leishmaniasis, prognosis, treatment

## Abstract

Granulomatosis with polyangiitis with nasal septal perforation can be confused with infectious diseases such as mucosal leishmaniasis, so these cases warrant an in‐depth study in order to provide the correct treatment. Among the main characteristics to consider to define a Wegener's granulomatosis as opposed to an infectious disease are vasculitis, lymphadenopathy, and sinusopathy.

## Graphical abstract

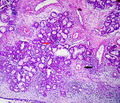

## 1 INTRODUCTION

We report a case of granulomatosis with polyangiitis, formerly called Wegener's granulomatosis, with differential diagnosis of mucosal leishmaniasis, which was the initial presumptive diagnosis. This report highlights the clinical characteristics such as vasculitis, lymphadenopathy, sinusopathy, pulmonary nodules, and the importance of performing a differential diagnosis in order to determine the correct treatment and prognosis in the early stage of the disease.

Granulomatosis with polyangiitis, formerly called Wegener's granulomatosis, is a systemic disease characterized by small‐ and medium‐sized vessel vasculitis.^1^, ^2^ The characteristic clinical triad consists of necrotizing granulomas of the upper and lower respiratory tract, systemic vasculitis, and necrotizing glomerulonephritis; however, this is only seen in limited forms of the disease.^1^ Only 11% of patients experience epistaxis as the initial clinical presentation. The most common clinical findings are upper respiratory tract involvement, sinusitis, and pulmonary symptoms.^3^ Most cases show positive C‐type antineutrophil cytoplasmic antibodies (ANCA), with a minority showing positivity for myeloperoxidase (MPO), while others are negative for both.^4^ Among the differential diagnoses which must be considered, especially in endemic areas, is mucocutaneous leishmaniasis.^5^

Leishmaniasis is a tropical disease showing worldwide distribution and caused by a parasite of the genus *Leishmania* ^6^ About 10% patients affected by the cutaneous form develop the mucocutaneous form, which causes damage to the nasal mucosa and which can spread to the oral mucosa, larynx, and nose.^6^ Since both granulomatosis with polyangiitis and mucocutaneous leishmaniasis affect the nasal mucosa and can be sometimes difficult to diagnose, it is important to make an accurate differentiation based on the clinical picture and with the support of laboratory tests.

Here, we report the case of a female patient whose first symptoms were chronic epistaxis and nasal septal perforation with a presumptive diagnosis of mucosal leishmaniasis, which was discarded based on laboratory and imaging tests, as well as her clinical characteristics. Following a review and critical discussion of the case, including her immune profile, a conclusive diagnosis of granulomatosis with polyangiitis was reached.

## 2 CASE PRESENTATION

This study included a thirty‐five‐year‐old female patient from Arequipa, Peru with a history of asthma; although her blood count showed 0.7% eosinophils (1‐4) and 0.10% basophils (0.20‐1.20). For over 3 years, she had experienced yellowish nasal discharge stained with blood (6‐10 episodes per month).

She was referred to Lima with a presumptive diagnosis of mucosal leishmaniasis based on the perforation of her nasal septum with bloody secretion, and her history of having traveled 9 years ago to the rural district area of Tauria, Arequipa, Peru where the patient resided for 15 days and where she was bitten by blood‐sucking insects. Indirect immunofluorescence (IIF) tests for *Leishmania* and the leishmanin test were negative. An initial biopsy showed acute and chronic inflammation of infectious etiology, and Giemsa staining showed *Staphylococcus aureus*. IIF for *Leishmania* and leishmanin tests were performed again but were both negative. A biopsy sample was taken from the nasal mucosa, and a molecular PCR test was performed to detect the *Leishmania* kinetoplast, which was also negative. Creatinine 0.59 mg/dL (NV 0.6‐1.1 mg/dL), Urea: 1.498 mmol/L (1.7‐6.7), 24h proteinuria 254.83 mg/24h (NV<150), and BUN 8.8 mg/dL (NV 9‐23).

**FIGURE 1:**
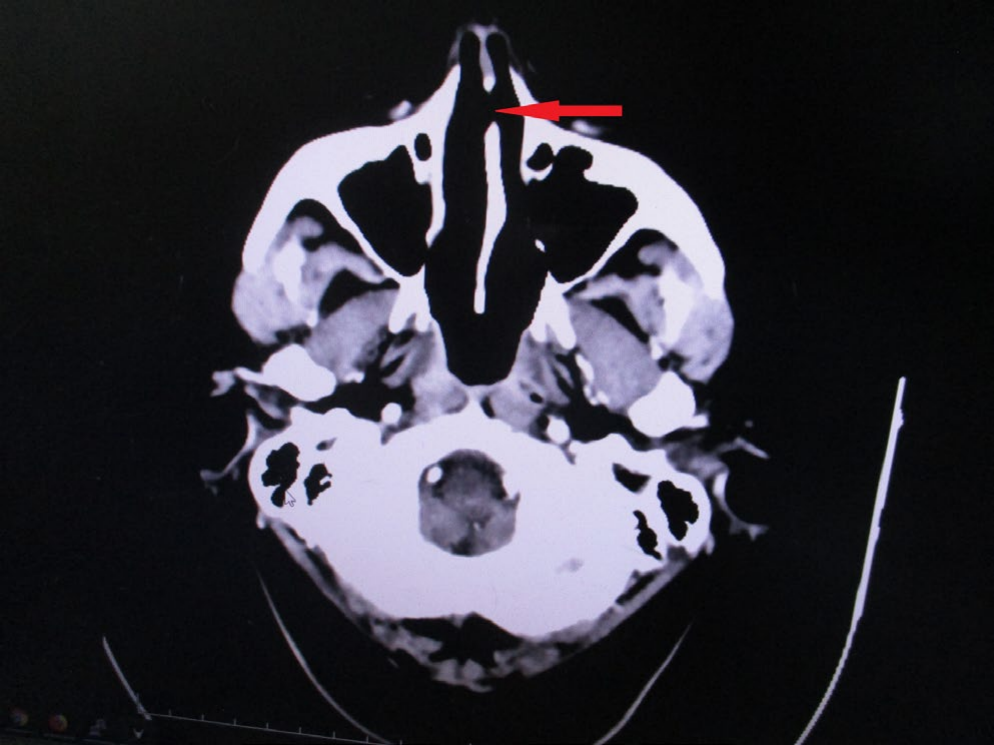
Head computed tomography of the patient, showing perforation of the cartilaginous part of the nasal septum (red arrow)

Discarding an infectious agent, we began to suspect an immune cause. Tests for antinuclear antibodies (ANA) and ANCA were negative. A computed axial tomography (CAT) showed mild bilateral maxillary sinusitis with deviation of the nasal septum to the left and showed perforation of the cartilaginous part of the nasal septum (Figure 1). The patient was not a cocaine user, ruling out the case and the histological findings considered in cocaine‐induced septum amputation.

**FIGURE 2:**
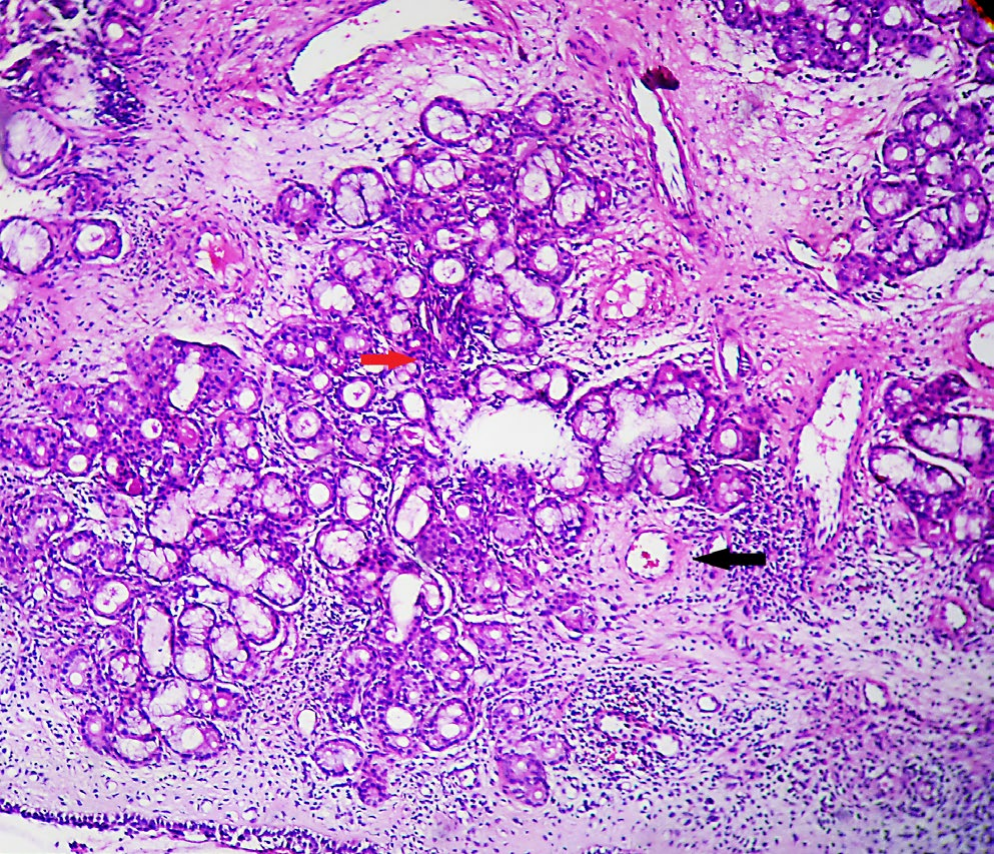
Moderate chronic inflammatory lymphoplasmacytic infiltration of the mucosa (red arrow). Abundant vessels with hyalinized walls and prominent endothelium in the submucosa (black arrow; 400X)

Two years and 3 months later, the IgE test showed levels of 1265 U/mL, eosinophils 1.9% (NV 1‐4), lymphocytes 23.5% (NV 17‐45), basophils 0.20% (NV 0.20‐1.20), monocytes 3.70% (NV 2‐8), segmented cells 70.6% (NV 55‐75), and a CD4/CD8 ratio of 2.30. The examination of IgG subclasses (IgG 1, IgG 2, IgG 3, and IgG 4) was performed with values within the normal range: 757 mg/dL, 349 mg/dL, 71.40 mg/dL, and 56.80 mg/dL, respectively. One year and 7 months later, three additional nasal mucosa biopsies were performed showing the following findings: mild stromal fibrosis, mild chronic inflammation, and vascular congestion (first biopsy), mild epithelial hyperplasia, stroma with fibrosis plus a few lymphocytes, and presence of seromucous glands with fibrosis and hemorrhages (second biopsy) and mild chronic superficial inflammation lymphoplasmacytic infiltration of the mucosa, edema, and vascular congestion abundant vessels with hyalinized walls and prominent endothelium in the submucosa without other alterations (third biopsy; Figure 2).

**FIGURE 3:**
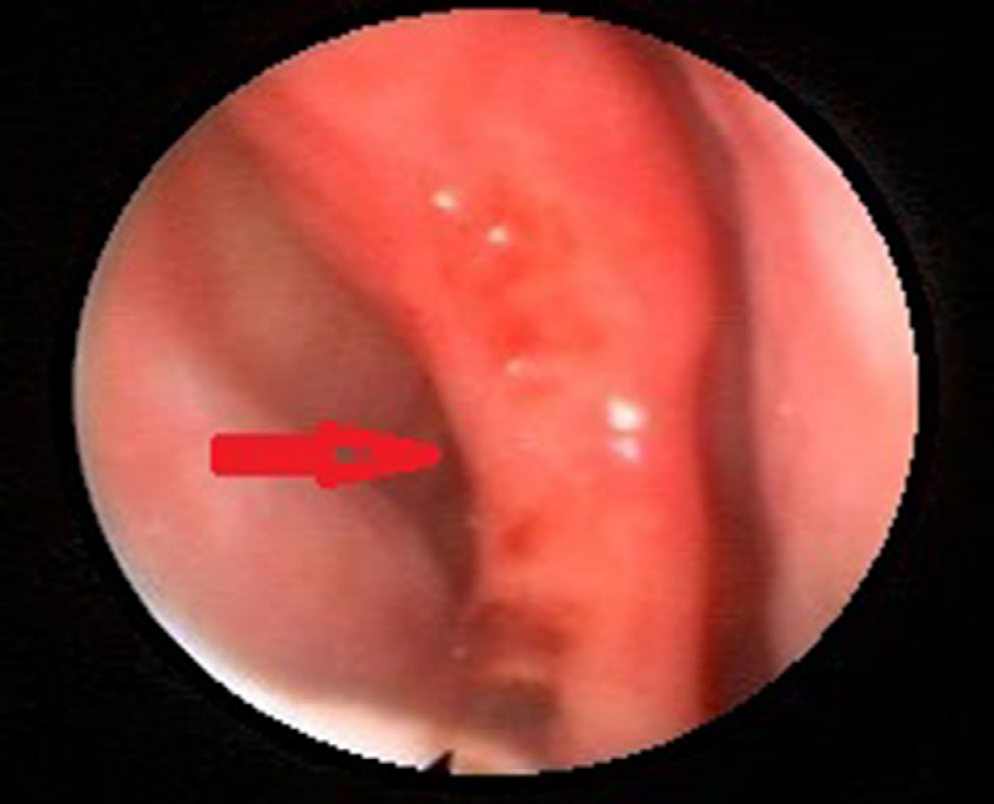
Middle third of the nasal septum in areas II and III, where the perforation of the nasal septum is observed (red arrow)

**FIGURE 4:**
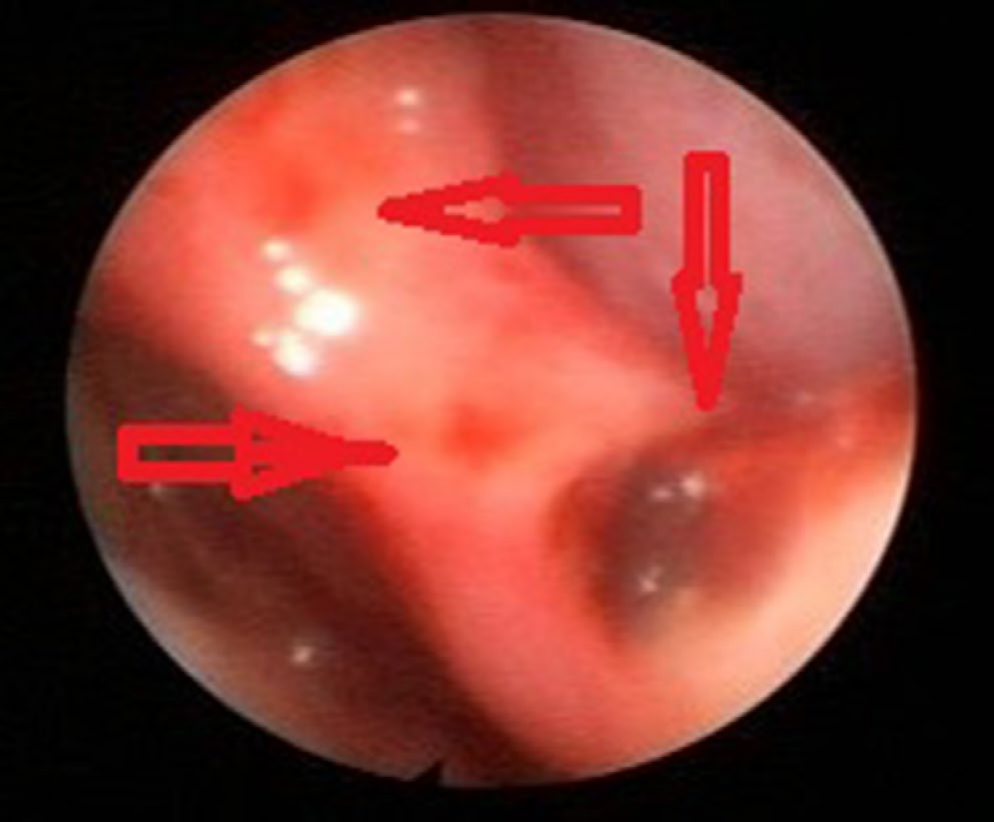
Middle third of the nasal septum in areas II and III, Middle third of the nasal septum in areas II and III, where areas of congestive mucosa, hemorrhagic points, and edema are observed (red arrows)

The patient was referred to an otorhinolaryngologist, who reported bilateral sensorineural hearing loss, predominantly of the right side. The rhinoscopy showed wide septal perforation of 3 cm in Cottle's areas II‐III with congestive mucosa, hemorrhagic points, and edema (Figures 3 and 4).

Ultrasound examinations showed a 1 cm tumor in the left breast with a 28 × 9 mm reactive lymph node and a 37 × 10 mm right axillary lymph nod, and non‐reactive lymph nodes in the lateral, inferior, and upper right cervical chains. CAT showed a pulmonary nodule with a diameter of 4 mm located in the right segment VI, suggestive of granuloma. A tomography of the chest with contrast showed mild congestion by decline and a small nodular lesion (3.4 mm) at the level of the segment VI of the right lower lobe, suggestive of granuloma. Maxillofacial and neck contrast tomography showed signs of right chronic maxillary sinusopathy, mild convex deviation of the nasal septum to the left, mild mucosal hypertrophy of the lower right turbinate, and signs of perforation of the nasal septum.

Continuing with the presumption of an underlying immune disease, duplicate venous blood samples were collected and subjected to cytokine analysis with Human Cytokine Magnetic 30‐Plex (Invitrogen^®^). Results were compared with control samples from a patient with no history of immune disease. Significant results for the analyzed cytokines were as follows: RANTES 2703 pg/mL ± 710.84 vs 161.8 pg/mL ± 84.39 for the control, *P* < .01; eotaxin 123.50 pg/mL ± 20.21 vs 44 pg/mL ± 13.72, *P* < .05; MCP‐1 171.80 pg/mL ± 1.35 vs 63.90 pg/mL ± 23.64, *P* < .01; IL‐1 RA 1380 pg/mL ± 52.17 vs 112.20 pg/mL ± 36.01, *P* < .01; IL‐8 126 pg/mL ± 6.12 vs 145.89 pg/mL ± 52.17, *P* = .73; G‐CSF 7.2 pg/mL ± 0.49 vs 133.80 pg/mL ± 52.17, *P* < .05; IL‐1 beta 9.20 pg/mL ± 0.49 vs 175 pg/mL ± 67.36, *P* < .05; and TNFα 5.60 pg/mL ± 0.25 vs 13.50 pg/mL ± 3.06, *P* < .05.

The patient was treated with methotrexate (7.5 mg every 12 hours, once per week), prednisone (20 mg at 8am and 10 mg at 4pm), delayed‐release omeprazole (20 mg before breakfast daily), folic acid (1 mg at breakfast, Monday to Saturday), sulfamethoxazole + trimethoprim (800 mg + 160 mg, respectively, one tablet at 8pm 4 times a week), and calcium carbonate (500 mg) + calcitriol (0.25 mg) daily at breakfast.

Currently, the patient has a diagnosis of granulomatosis with polyangiitis, breast duct ectasia, and cervical adenomegaly; she is stable and receives corticosteroid therapy.

## 3 DISCUSSION

The initial biopsy coincided with an infectious cause, showing *S aureus* in the nasal mucosa, associated with a diagnosis of granulomatosis with polyangiitis (GPA). It has been reported that this bacteria can affect the nasal mucosa and trigger a relapse due to a decrease in IL‐8 and G‐CSF,^7^ although the bacteria itself is not associated with the patient's systemic clinical picture.^7^, ^8^

A previous study indicated that patients suffering from granulomatosis with polyangiitis (GPA) have significantly decreased IL‐8 and G‐CSF levels.^7^ This is partly consistent with our study, since G‐CSF levels were significantly decreased, although IL‐8 showed no significant difference.^7^ Therefore, little neutrophil proliferation is expected in patients with GPA. In contrast, *Leishmania* infection induces the release of IL‐8, which is a chemokine that attracts polymorphonuclear cells to the site of infection and plays an important role in neutrophil recruitment and activation of cellular immunity.^9^

Therefore, as described above, in the case of levels of RANTES, eotaxin, and MCP‐1 pro‐inflammatory cytokines would not be sufficient to discriminate between a disease caused by an infectious agent like *Leishmania* and granulomatosis with polyangiitis. ^10^, ^11^, ^12^ In contrast, in an infection by *L*. *braziliensis*, which is the main species causing nasal damage, higher levels of TNFα would be expected when compared to those found in granulomatosis with polyangiitis, and this is consistent with what we observed in our study.^5^, ^7^

A previous study showed the importance of correctly discriminating between granulomatosis with polyangiitis and leishmaniasis and that the correct treatment and prognosis of the patient will depend on an accurate diagnosis.^5^ That study described the case of a patient with mucocutaneous leishmaniasis which masqueraded as granulomatosis with polyangiitis due to the similarity of the clinical picture.^5^ In contrast, in our case the patient was initially presumed to suffer from mucous leishmaniasis, but this was discarded and it was concluded that the correct diagnosis was granulomatosis with polyangiitis. On the other hand, a diagnosis of GPA requires a combination of clinical criteria and laboratory tests.^13^ Our study highlights the clinical characteristics such as lymphadenopathy, sinusopathy, and pulmonary nodules.

In the differential diagnosis of granulomatosis with polyangiitis, one should also consider different infectious and neoplastic diseases that can damage the mucous membranes, as can be seen in mucocutaneous leishmaniasis, paracoccidioidomycosis, tuberculosis, sporotrichosis, and malignancies.^14^

Chemokines such as RANTES (CCL5) and eotaxin (CCL11) are powerful chemoattractants for lymphocytes and macrophages, as well as for eosinophils and dendritic cells, respectively, to the site of inflammation. High levels of these chemokines have been reported in lung lesions associated with granulomatosis with polyangiitis.^15^ In the case of MCP‐1, which is a chemoattractant for monocytes, contradictory results have been reported in granulomatosis with polyangiitis when comparing the levels with those of healthy controls. In some cases, increased MCP‐1 levels have been observed in patients with this disease.^16^ However, other studies have found an inverse relationship between MCP‐1 levels and disease activity.^17^ In our study, these proinflammatory chemokines were elevated and this was consistent with the immunopathological picture of polyangiitis. Although microscopically macrophages and lymphocytes were present, at the tissue level, no eosinophils were observed. An interesting finding was significantly high IL‐1RA levels, implying a significant decrease in TNFα and IL‐1 levels.^18^ This was observed in our study, which would mean a relative inhibition of granulomatous processes at the tissue level in this chronic inflammatory condition.^19^ In addition, pulmonary nodules and adenopathy were observed at the cervical level, as reported in another study.^20^

It should be noted that although a positive ANCA test and a granulomatous clinical picture are expected in polyangiitis, these are not seen in all cases, as shown in our study. In this regard, it is sometimes difficult to take a biopsy from the affected tissue at the right time and site to find the granulomas.^21^ In our study, except for bilateral lithiasis, no renal involvement was observed in the patient, who had normal levels of creatinine and urea. The characteristics of granulomatosis with polyangiitis of our patient coincided with those reported in previous studies: sinusitis, rhinorrhea, nasal scabs, nasal septal perforation, and sensorineural hearing loss.

## 4 CONCLUSIONS

It can be concluded that the clinical presentation of granulomatosis with polyangiitis with nasal septal perforation can be confused with infectious diseases such as mucosal leishmaniasis, so these cases warrant an in‐depth study of the clinical picture, supported by laboratory tests. In our study, granulomatosis with polyangiitis was defined as ANCA negative, without the presence of granulomas in nasal mucosal tissues, and with significantly high levels of RANTES, eotaxin, MCP‐1, and IL‐1 RA.

## CONFLICT OF INTEREST

None declared.

## AUTHOR CONTRIBUTIONS

JR‐J: involved in conception of idea, manuscript writing, and final approval. VHR‐F: involved in acquisition of data, manuscript writing, and final approval. RC and JA: involved in critical revision, manuscript writing, and final approval. JG‐R: involved in manuscript writing and final approval.

## ETHICAL APPROVAL

This manuscript did not receive any financial support.

## Data Availability

The data will be made available on request due to ethics and privacy.
